# The definition of sexual selection: a response to comments on Shuker and Kvarnemo

**DOI:** 10.1093/beheco/arab085

**Published:** 2021-08-09

**Authors:** David M Shuker, Charlotta Kvarnemo

**Affiliations:** 1 School of Biology, University of St Andrews, Harold Mitchell Building, St Andrews KY16 9TH, UK; 2 Department of Biological and Environmental Sciences, University of Gothenburg, Gothenburg, Sweden

We are very grateful for the seven commentaries on Shuker and Kvarnemo (2021) and the breadth of discussion they bring. Unfortunately, we cannot do justice to each piece and will instead focus on one over-arching aspect of that discussion, the value of a robust concept of sexual selection. That said, we agree wholeheartedly with Andersson (2021), and perhaps contra to Clutton-Brock (2021), that the term “inter-sexual selection” should be retired.

Alonzo and Servedio (2021) emphasize the important distinction between conceptualizing and operationalizing sexual selection. As they note, our paper focuses on the former, but it does so because we feel that how to operationalize our definition is already well-embedded in behavioral ecology. Via measures of selection, in both the field and the laboratory, under both natural and experimental conditions, behavioral ecologists have long been adept at bringing the relevant statistical tools—including those highlighted by Shuster and Wade (2021)—to the study of traits thought to be under sexual selection. What we hope we have achieved here is to clarify the *nature* of sexual selection as a fitness component. As such, we reaffirm the need for careful conceptualization, alongside the clear desire for measuring sexual selection so strongly advocated by Shuster and Wade, because measurements and observations—the data empiricists all thrive on—are of little value without *interpretation*. The science of evolutionary biology is in that interpretation. And as Simmons and Parker (2021) caution, interpretations can change, as new phenomena are discovered.

Clutton-Brock (2021) reiterates our point that some cases of sexual selection will need to be re-evaluated. But we think it is important that behavioural ecologists use a definition that is conceptually robust, with a clear logic. Without that clear logic, we are not sure that it will be easier to explain sexual selection to the general public. Similarity of form needs not necessarily mean similarity of function. For instance, organisms can fight for different things, males and females alike. We need to recognise that, and not call all selection on weapons one thing, just because they are weapons.

In terms of the broader point made by Alonzo and Servedio (see also Alonzo and Servedio 2019), we do not so much disagree as to there being gray areas in sexual selection, rather it is where those gray areas are. We continue to think that conceptually sexual selection does not have gray areas: that is why we wrote a one sentence definition of it. However, operationally we fully concur that there are gray areas, more than fifty shades of them perhaps. As we emphasized, it will be hard to identify and quantify sexual selection on a trait in numerous real-life cases. Different fitness components may align. In her commentary, Kokko (2021) provides a characteristically clear-sighted discussion of this point. But, we also feel that sexual selection should not be held to a higher standard. For instance, conceptually there is perhaps little fuss over viability selection (selection via survival) or fecundity selection (selection via, well, fecundity). However, to operationalize those two components of fitness empirically is also difficult in real life. That might mean that there are times when we put such delineation of fitness components to one side (see also Shuker 2010), but being aware that fitness can vary thanks to viability, fecundity, or competition for access to gametes, has conceptual value, and brings interpretation to our data.

The empirical measurement of sexual selection is the focus of Shuster and Wade’s commentary, work that remains at the heart of our field. However, perhaps that focus has led those authors to consider our definition as overly narrow. In contrast, we agree wholeheartedly with Zuk (2021) in thinking that our definition is exceptionally *broad*. Deliberately, we do not tie sexual selection to any given mechanism (such as mate choice), nor to any sex or sex role, nor indeed to anisogamy or isogamy. We agree with Simmons and Parker (2021) that anisogamy—the generation of two sexual functions—has had a remarkable impact on organismal evolution, as captured by the “sexual cascade” of Parker (2014; Parker and Pizzari 2015). But, the focus on gametes—anisogamous or not—in fact allows the broadest range of mechanisms to impinge on sexual selection, from meiotic drive to mating displays. In that sense, we leave the operationalization of sexual selection up to nature.

Finally, we also do not exclude indirect genetic effects (IGE) nor multi-level selection. After all, mate choice—while not originally conceptualized that way—is a quintessential IGE, with two classes of social actors (males and females) and an interaction coefficient (ψ, or “mate preference” in more usual terminology). Beyond individuals, the fact that groups of same-sex individuals may cooperate, and be more successful in gaining access to opposite-sex gametes than individuals acting on their own are, is also in no way excluded from our definition of sexual selection (see also Shuker 2010). We might disagree as to whether such group courtship or coercion is a “group adaptation,” or instead a strategy by which individuals cooperate with each other to maximize their inclusive fitness, but the mathematics end up the same.

In conclusion, we agree with many of the comments that the future will no doubt bring many new empirical challenges for students of sexual selection, but we hope our definition provides a strong starting point for meeting those challenges.
